# Functional Morphology of the Arm Spine Joint and Adjacent Structures of the Brittlestar *Ophiocomina nigra* (Echinodermata: Ophiuroidea)

**DOI:** 10.1371/journal.pone.0167533

**Published:** 2016-12-14

**Authors:** Iain C. Wilkie

**Affiliations:** Department of Life Sciences, Glasgow Caledonian University, Glasgow G4 0BA, Scotland, United Kingdom; Laboratoire de Biologie du Développement de Villefranche-sur-Mer, FRANCE

## Abstract

The skeletal morphology of the arm spine joint of the brittlestar *Ophiocomina nigra* was examined by scanning electron microscopy and the associated epidermis, connective tissue structures, juxtaligamental system and muscle by optical and transmission electron microscopy. The behaviour of spines in living animals was observed and two experiments were conducted to establish if the spine ligament is mutable collagenous tissue: these determined (1) if animals could detach spines to which plastic tags had been attached and (2) if the extension under constant load of isolated joint preparations was affected by high potassium stimulation. The articulation normally operates as a flexible joint in which the articular surfaces are separated by compliant connective tissue. The articular surfaces comprise a reniform apposition and peg-in-socket mechanical stop, and function primarily to stabilise spines in the erect position. Erect spines can be completely immobilised, which depends on the ligament having mutable tensile properties, as was inferred from the ability of animals to detach tagged spines and the responsiveness of isolated joint preparations to high potassium. The epidermis surrounding the joint has circumferential constrictions that facilitate compression folding and unfolding when the spine is inclined. The interarticular connective tissue is an acellular meshwork of collagen fibril bundles and may serve to reduce frictional forces between the articular surfaces. The ligament consists of parallel bundles of collagen fibrils and 7–14 nm microfibrils. Its passive elastic recoil contributes to the re-erection of inclined spines. The ligament is permeated by cell processes containing large dense-core vesicles, which belong to two types of juxtaligamental cells, one of which is probably peptidergic. The spine muscle consists of obliquely striated myocytes that are linked to the skeleton by extensions of their basement membranes. Muscle contraction may serve mainly to complete the process of spine erection by ensuring close contact between the articular surfaces.

## Introduction

The spininess of sea-urchins and their relations is celebrated in the name of their parent phylum–Echinodermata (‘hedgehog-skinned’). Calcified spines connected to the endoskeleton of the body wall at mobile articulations are a prominent feature of three of the five extant echinoderm classes—Asteroidea, Echinoidea and Ophiuroidea [1,2], and may have occurred in extinct members of another class (Crinoidea: [3]). As well as having a variety of defensive functions, echinoderm spines can be involved in locomotion, feeding, respiration and reproduction, in most of which roles the mobility of the spine joint, allowing spine inclination to be altered, is a critical attribute.

The functional morphology and mechanics of echinoid spine joints have been studied extensively [4–11]. Much less information is available on those of asteroids [12,13]. Whilst the general structure of ophiuroid spine joints had previously attracted only sporadic attention [14–16], there has been in recent years a proliferation of descriptive data on specific aspects of their skeletal morphology, which has been provoked by their potential taxonomic and palaeontological significance, and which has revealed a remarkable diversity across the class [17–25]. Although the functional importance of ophiuroid arm spines has been recognised [18], there is still a dearth of information on (1) the soft tissue components of the spine joints and (2) the functional significance of their skeletal morphology, which impedes a full understanding of the biological roles of the spines and depreciates the information content of ophiuroid fossils [20,21]. This stands in contrast to the considerable attention that has been paid to the functional morphology of the vertebral joints of ophiuroid arms [26–31].

**Fig 1:**
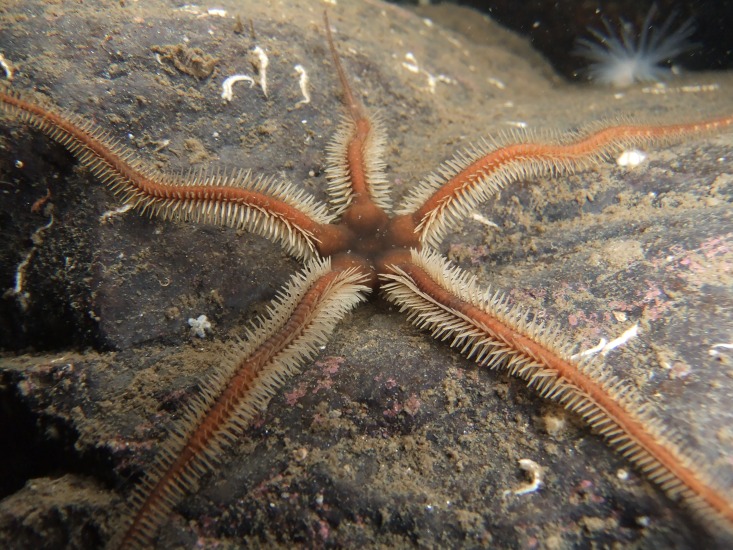
A specimen of *O*. *nigra* photographed *in situ* in Loch Leven, Scotland. (Photograph: James Lynott)

The present paper focuses on the arm spine joint of the common NE Atlantic and Mediterranean ophiuroid *Ophiocomina nigra* (Abildgaard in O.F. Müller, 1789) (Fig 1). It provides a detailed description of the skeletal morphology of the joint and the histological and ultrastructural organisation of its main soft tissue components, *viz*. the epidermis, connective tissue structures, juxtaligamental system and muscle. Particular attention was paid to the juxtaligamental node of the spine ligament, since the literature contains conflicting interpretations of the anatomical relations and structure of this node in other ophiuroid species. To help elucidate the functional significance of the spine joint, observations were made on the behaviour of the spines in intact living animals. Aspects of this behaviour, which indicated that the joint can display variable resistance to imposed movement, as well as certain morphological features, suggested that the spine ligament is a mutable collagenous structure, i.e. that its mechanical properties can change rapidly under nervous control [32–34]. This hypothesis was tested in an experiment on whole animals designed to ascertain if the spines can be detached completely (i.e. autotomised) in response to external stimulation, and in another experiment in which the effect of stimulation on the mechanical properties of individual spine joints was determined. Information provided in this paper shows that, far from being the simple articulations depicted in many diagrammatic representations of ophiuroid anatomy (see, e.g. [1,2]), ophiuroid arm spine joints can have a complex morphology, which, in the case of *O*. *nigra*, is partly related to the ability of the joint to switch between immobile and mobile states.

## Materials and Methods

### Animals

Specimens of *O*. *nigra* were obtained from various littoral and sublittoral sites around the Isle of Cumbrae, Firth of Clyde, Scotland by shore collection, SCUBA diving or dredging. They were held in tanks of aerated seawater at 8°C. *O*. *nigra* is not an endangered or protected species. Specific permission was not required for collection of the animals, since this took place on Crown property and is regarded as a tolerance.

### Light microscopy

Pieces of arm were fixed in Gomori's formol sublimate or Bouin’s fluid [35], decalcified in 3% nitric acid in 70% ethanol, and embedded in paraffin wax. Transverse and frontal (horizontal) sections 8 μm thick were stained with Milligan's trichrome, which provides good differentiation between basic tissue types, or Bargmann's chrome alum haematoxylin and phloxine, which facilitates identification of basement membranes and echinoderm juxtaligamental cells [35]. They were photographed in an Olympus BH-2 microscope equipped with a PM-10ADS camera system (Olympus UK Ltd., Southend-on-Sea, UK).

### Scanning electron microscopy

Soft tissues were removed wholly or partly from arm pieces using a solution of sodium hypochlorite (i.e. bleach). The specimens were then washed thoroughly, dehydrated in an ethanol series and in air, sputter-coated with gold, and observed in a Philips 500 SEM (FEI, Eindhoven, The Netherlands).

### Transmission electron microscopy

Lateral arm plates to which spines were attached were excised carefully from animals anaesthetised in seawater containing 0.1% propylene phenoxetol (1-phenoxy-2-propanol). The plates were fixed in 2% glutaraldehyde in seawater for 2 h, rinsed in seawater alone for 2 h, and post-fixed in 1% osmium tetraoxide in seawater for 2 h. They were decalcified in saturated ethylenediaminetetraacetic acid (pH adjusted to 7.0 with potassium hydroxide pellets) and embedded in Araldite. Ultrathin transverse sections were stained with uranyl acetate and lead citrate, and viewed in AEI EM801 (AEI Scientific Apparatus Ltd., Harlow, UK) and FEI Morgagni 268 (FEI, Eindhoven, The Netherlands) microscopes.

### Observations of spine behaviour in living animals

Most of the information on spine behaviour was obtained from six animals (disc diameter 10–15 mm), which were observed individually in 10 cm square petri dishes containing seawater using a Leica Wild M3Z zoom stereomicroscope (Leica Microsystems (UK) Ltd., Milton Keynes, UK). Spine behaviour was compared before and whilst the animals were anaesthetised by immersion for at least 30 min in seawater containing 0.1% propylene phenoxetol, and was also observed after autotomy induced by compressing arms near the disc with forceps.

### Spine tagging experiment

The aim of the tagging experiment was to test the hypothesis that *O*. *nigra* can detach arm spines by an intrinsic mechanism. It was anticipated that, if *O*. *nigra* has this capacity, it would be invoked by tags attached to spines, due to the irritant effect of the tags. Five animals (disc diameter 10–12 mm) were anaesthetised in 0.1% propylene phenoxetol in seawater for at least 20 min. Each animal was removed from the anaesthetic solution, placed on absorbent paper to remove excess moisture and a tag was attached to one spine on each of its five arms. The tags were plastic cubes of edge length 1 mm and mean weight 3.6 mg. One tag was fixed with cyanoacrylate cement to the outer end of a single spine located about halfway along the length of each arm. Each animal was then transferred to a separate tank containing 2 L of aerated seawater at 8°C. The tanks were inspected daily in order to record the date on which each tag was jettisoned.

### Experiment on individual spine joints

**Fig 2:**
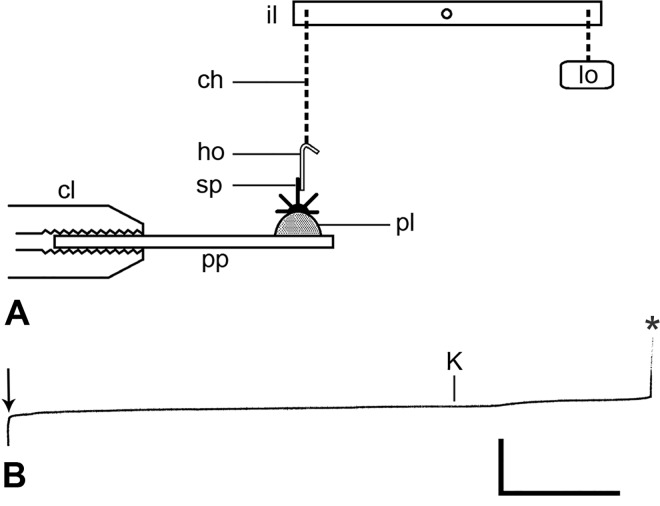
Experiment on individual spine joints. (A) Diagrammatic side-view of the apparatus (not to scale). ch, chain; cl, clamp; ho, hook; il, isotonic lever; lo, load; pl, Plasticine™; pp, plastic platform; sp, arm spine. (B) Representative recording of one test: after application of the load (arrow), the preparation was left undisturbed for 3.5 min then stimulated with seawater containing 100 mmol l^-1^ K^+^ ions (K), which was followed by an increase in the extension rate and rupture of the spine joint (asterisk). Horizontal scalebar: 1 min; vertical scalebar: 1 mm.

If the spine ligament is a mutable collagenous structure, it would be expected that its mechanical properties, and therefore those of the spine joint as a whole, would change following exposure to an elevated potassium ion concentration (which acts as a non-specific neurostimulant), as has been observed consistently in all echinoderm mutable collagenous structures that have been thus tested to date (see e.g. [8,36–38]). The responsiveness of the spine ligament to elevated [K^+^]_o_ was determined using preparations made from autotomised arms of two animals (disc diameter 15 mm), which were obtained by compressing the arms near their base with forceps. The proximal third of each arm was cut transversely by scalpel into pieces consisting of two segments, which were left in seawater for at least 30 min before further processing. Each arm-piece was then placed on filter paper to remove surface moisture and a hook made from tinned copper wire (diameter 0.2 mm) was attached to a spine with cyanoacrylate cement (Fig 2A). The arm-piece, orientated with the aforementioned spine projecting upwards, was fixed to a 1mm thick plastic platform with Plasticine™ and the platform was held horizontally in the jaws of a clamp (Fig 2A). The hook was connected to one end of an isotonic lever (Harvard Apparatus, Cambridge, UK) by means of a silver jewellery chain and from the other end of the lever a weight was suspended, which subjected the spine to a constant tensile load of 1.2 gf. Output from the isotonic transducer was fed to an oscillograph (Harvard Apparatus, Cambridge, UK), which produced a recording of change in length (extension) against time (Fig 2B). Before a test started, the weighted end of the lever rested on a horizontal rod attached to a manipulator, so that no tension was applied to the spine. The manipulator was then used to lower the lever slowly until the spine was subjected to the full tensile load. Preparations were left undisturbed for at least 3 min before they were flooded with 0.56 mol l^-1^ KCl solution (which is isosmotic with seawater) or with high-potassium seawater containing 100 mmol l^-1^ K^+^ delivered from a syringe. The experiment was conducted at 10°C.

## Results

### Background anatomy

**Fig 3:**
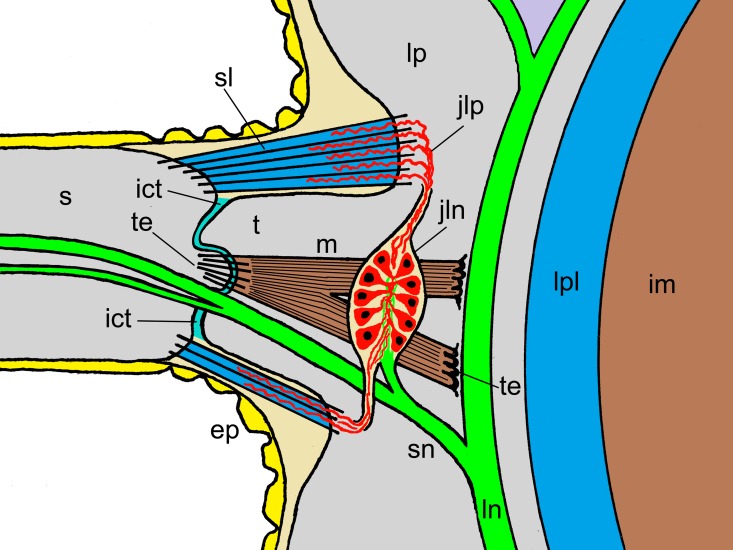
Main anatomical components of the arm spine joint. Diagrammatic representation of part of a transverse section of the arm of *O*. *nigra* showing one arm spine joint. This is not to scale and is a simplified and idealised depiction of anatomical relationships, since in a real animal not all the components would be included in the same plane of section. ep, epidermis; ict, interarticular connective tissue; im, intervertebral muscle; jln, juxtaligamental node; jlp, juxtaligamental cell processes; ln, lateral arm plate nerve; lp, lateral arm plate; lpl, ligament connecting adjacent lateral arm plates; m, spine muscle; s, spine; sl, spine ligament; sn, spine nerve; t, tubercle; te, tendons.

**Fig 4:**
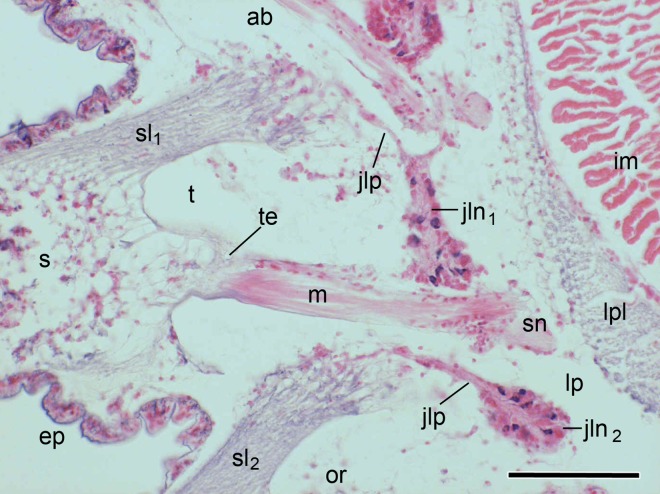
Main anatomical components of the arm spine joint. Transverse histological section showing actual anatomical relationships. ab, aboral (dorsal) side; ep, epidermis; im, intervertebral muscle; jln_1_, juxtaligamental node associated with the ligament of the featured joint; jln_2_, juxtaligamental node associated with the ligament of the adjacent joint; jlp, juxtaligamental cell processes; lp, lateral arm plate; lpl, ligament connecting adjacent lateral arm plates; m, spine muscle; or, oral (ventral) side; s, spine; sl_1_, ligament of featured joint; sl_2_, ligament of adjacent joint; sn, spine nerve (sectioned obliquely); t, tubercle; te, tendon. Scalebar: 0.1 mm.

**Fig 5:**
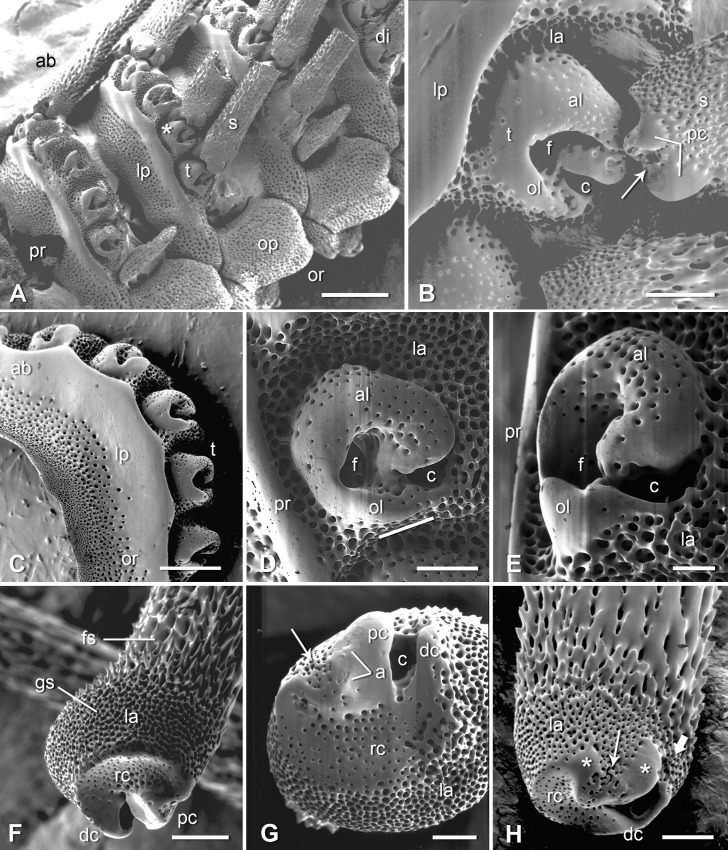
Scanning electron micrographs of the skeletal components of the arm spine joint. (A) Lateral view of three segments of an incompletely digested arm. The asterisk marks the joint shown at higher magnification in B. Scalebar: 0.5 mm. (B) More magnified view of the disarticulated joint marked by an asterisk in A. Scalebar: 0.1 mm. (C) Isolated lateral arm plate. View of proximal side showing tubercles. Scalebar: 0.25 mm. (D) Lateral view of a tubercle. The line below the oral lobe shows the approximate position of the gap in the insertion area of the ligament associated with the illustrated tubercle. Scalebar: 0.1 mm. (E) Distal view of a tubercle. Scalebar: 50 μm. (F) Adradial end of a detached spine: oblique view of aboral and articular surfaces. Scalebar: 0.1 mm. (G) Articular surface of a spine. Scalebar: 50 μm. (H) Adradial end of a detached spine: oblique view of oro-proximal and articular surfaces. The asterisks mark the proximal (left) and distal (right) imperforate regions of the proximal condyle. Scalebar: 0.1 mm. a, abrasions; ab, aboral (dorsal) side; al, aboral lobe of tubercle; c, opening of nerve canal; dc, distal condyle; di, distal (nearer arm-tip) end; f, muscle fossa; fs, fascicular stereom; gs, galleried stereom; la, ligament insertion area; lp, lateral arm plate; ol, oral lobe of tubercle; op, oral arm plate; or, oral (ventral) side; pc, proximal condyle; pr, proximal (nearer central disc) end; rc, reniform concavity; s, spine; t, tubercle; thin arrow, muscle insertion area; thick arrow, position of gap in ligament insertion area.

The anatomy of the spine joint and adjacent structures is illustrated in Figs 3 and 4. The main skeletal components of each arm segment of *O*. *nigra* are an internal vertebral ossicle and four external arm plates: aboral (dorsal), oral (ventral) and paired lateral plates. Each lateral arm plate carries a vertical row of five to seven spines (Fig 5A). The outermost tissue of the spines and external arm plates is an epidermis. Each spine is connected to the lateral arm plate by a ligament that almost completely encircles the joint and by an eccentrically placed muscle. The spine contains two prominent axially orientated nerves, which start as a single branch of a large nerve that ascends within the lateral arm plate and itself originates as a segmental branch of the radial nerve cord in the oral side of the arm. The undivided spine nerve bifurcates within the lateral arm plate near the articulation with the spine (Fig 3).

### Morphology of skeletal components

Each spine is attached to an articular tubercle on the exterior lateral surface of a lateral arm plate (Fig 5A) (The term ‘tubercle’ is used herein in preference to the more cumbersome ‘arm spine articulation’ advocated by Stöhr et al. [25]). The tubercle has the form of a roughly circular ridge of varying height and lateral thickness surrounding a deep fossa that houses the spine muscle. The oro-distal region of the ridge is interrupted by a slit-shaped gap, which is the external opening of the spine nerve canal. The aboral (dorsal) lobe of the ridge is a wide, reniform (kidney-shaped) convexity; the oral (ventral) lobe is narrower and has a sharper apex. The calcite stereom of the whole tubercle is sparsely perforate, though that of the oral lobe is almost imperforate, especially on its oral-facing side (Fig 5A–5E).

The stereom of the lateral arm plate at the base of each tubercle is of the galleried type to which is attached the spine ligament. The insertion area of the ligament (and therefore the ligament itself) does not completely surround the tubercle: there is a wide gap at the oro-proximal side adjacent to the oral lobe (Fig 5D).

**Fig 6:**
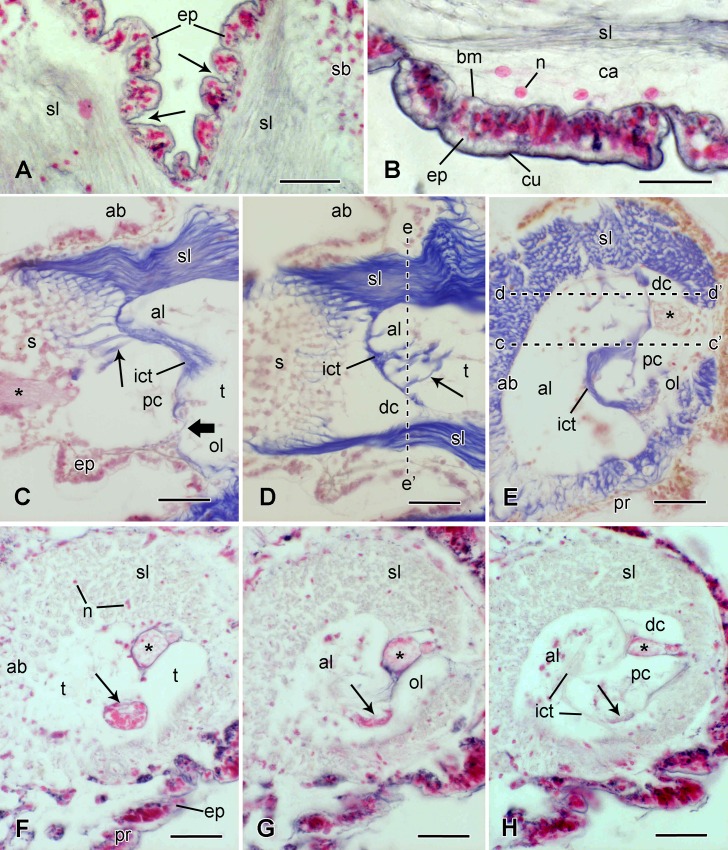
Microstructure of soft tissue components of the joint. Light micrographs. (A, B) Circumarticular epidermis. Transverse sections stained with Bargmann’s chrome alum haematoxylin and phloxine (CAHP: connective tissue grey) showing epidermal constrictions (arrows). Scalebars: 40 μm (A) and 20 μm (B). (C, D) Spine joint, the spine being in the fully erect position. Two sections from the same series, stained with Milligan’s trichrome (MT: connective tissue blue). The plane of these sections is vertical and parallel to the longitudinal axis of the spine (which projects to the left); their location is shown by the dashed lines in E. The thin arrows indicate fibres connecting the interarticular connective tissue to the skeleton, and the thick arrow indicates the area of direct contact between the proximal condyle and oral lobe of the tubercle. Note that the spine ligament is not visible at the oral side of the joint in C, because the section plane passes through the ligament gap. Scalebars: 40 μm. (E-H) Spine joint. Transverse sections through the tubercle in plane perpendicular to the longitudinal axis of the erect spine. (E) Stained with MT. Plane of section shown in D. Note the gap in the oral side of the ligament (between c’ and d’). Scalebar: 40 μm. (F-H) Stained with CAHP. Three sections from the same series, the plane of section being progressively abradial (towards the spine and away from the lateral arm plate). (F) Plane of section is below the tubercle lobes. The muscle (thin arrow) occupies a wide fossa. The spine nerve (asterisk) has bifurcated into larger and smaller branches, which are still contiguous. The ligament contains sparse cell bodies, as revealed by their nuclei. Scalebar: 40 μm. (G) Plane of section is below the spine base, but the tubercle lobes are visible (al, ol). The arrow indicates the spine muscle. Scalebar: 40 μm. (H) Plane of section is at about same level as in E (see ee’ in D) and includes both tubercle and spine base. The arrow indicates the abradial edge of the spine muscle insertion area. Scalebar: 40 μm. Asterisk, spine nerve; ab, aboral side; al, aboral lobe of tubercle; bm, basement membrane; ca, cavity; cc’ and dd’, planes of sections shown in C and D respectively; cu, cuticle; dc, distal condyle of spine base; ee’, plane of section shown in E; ep, epidermis; ict, interarticular connective tissue; n, nucleus; ol, oral lobe of tubercle; pc, proximal condyle of spine base; pr, proximal side; s, spine; sb, spine base; sl, spine ligament; t, tubercle.

The articular surface of the spine base consists of a reniform concavity of sparsely perforate stereom, which, when the spine is erect, sits against the aboral lobe of the tubercle, and two condyles, which are located orally on either side of the opening of the spine nerve canal. The distal condyle is smaller and its outer surface is imperforate. The much larger proximal condyle consists of distal and proximal regions of imperforate stereom, between which on its outer oral-facing side there is a shallow depression of perforate stereom, which is the insertion area of the spine muscle (Fig 5F–5H). When the spine is fully erect, both condyles fit against the aboral lobe of the tubercle (Fig 6C–6E) and the proximal condyle contacts the oral lobe of the tubercle (Fig 6C); the condyles thereby act as a mechanical stop that prevents inclination of the spine in an oral or oro-proximal direction. Beyond the articular area of the spine base is a collar of galleried stereom to which the abradial end of the spine ligament is attached and which contrasts with the fascicular stereom of the rest of the spine beyond the articulation complex (Fig 5F and 5H). There is an interruption in the ligament insertion area at the oro-proximal side of the spine base above the gap between the distal and proximal condyles (Fig 5H).

Although few surface abrasions attributable to mechanical wear were observed on the articular surfaces of the tubercle and spine base (see [39]), there was consistent damage to the distal region of the proximal condyle of the spine base near the muscle insertion area, which was apparent in four separate preparations (Fig 5G). This region of the proximal condyle fits against the oral lobe of the tubercle when the spine is fully erect (Fig 6C).

### Histology and ultrastructure of soft tissue components

#### Circumarticular epidermis

The generalised ophiuroid epidermis consists of an outer extracellular cuticle, a middle cellular layer, and an inner basement membrane comprising a lamina lucida and lamina densa (Fig 6A and 6B). The most numerous components of the cellular layer are “support” cells that extend from the cuticle (which is penetrated by their apical microvilli) to the basement membrane. Other cells, including pigment cells, secretory cells and basiepithelial neural elements, are usually present. Over most of the external arm plates and spine shaft the epidermis is contiguous with the underlying skeleton and attached directly to it, for example through groups of epidermal cells located in stereom spaces [16]. The histology and ultrastructure of this tightly anchored type of epidermis in *O*. *nigra* have been described in detail elsewhere [40–42].

**Fig 7:**
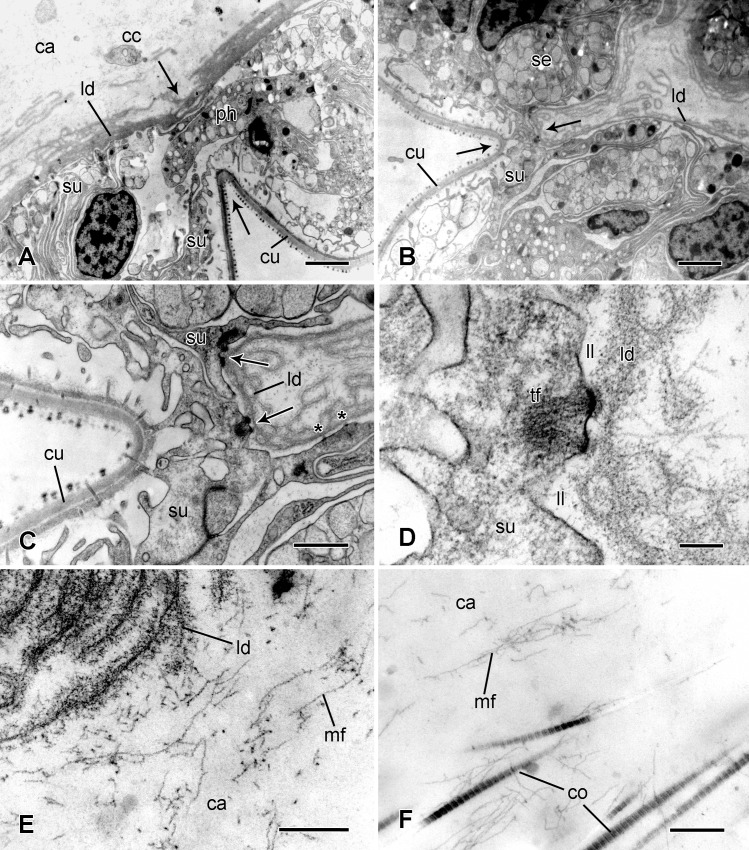
Ultrastructure of the circumarticular epidermis. Transmission electron micrographs. (A) Epidermal constriction (between arrows). Scalebar: 2 μm. (B) Extreme epidermal constriction (between arrows). Scalebar: 2 μm. (C) More magnified view of epidermal constriction shown in B. The arrows indicate large hemidesmosome-like junctions between the lamina densa and the support cells. The asterisks mark small hemidesmosome-like junctions. Scalebar: 1 μm. (D) Large hemidesmosome-like junction (indicated by lower arrow in C). Scalebar: 0.2 μm. (E) Edge of lamina densa next to cavity between it and spine ligament, showing delaminated appearance. Scalebar: 0.2 μm. (F) Edge of spine ligament next to cavity. Scalebar: 0.5 μm. ca, cavity between epidermis and spine ligament; cc, possible coelomocyte; co, collagen fibrils; cu, cuticle; ld, lamina densa; ll, lamina lucida; mf, microfibrils; ph, possible phagocyte; se, secretory cell; su, support cell; tf, tonofibrils.

At the spine joint the epidermis is not tightly anchored to underlying skeletal elements. It overlies the spine ligament and is separated from it by a cavity (up to 15 μm wide in material prepared for TEM) containing sparse microfibrils (diameter around 10 nm) and a few unattached cells, which are probably different coelomocyte types (Figs 6A, 6B, 7A, 7E and 7F); there is no trace of a differentiated boundary layer (cellular or extracellular) separating the cavity from the collagen fibrils of the ligament (Fig 7F). In intact arms the epidermis surrounding the joint has a wrinkled appearance, which results from the presence of narrow circumferential constrictions (Fig 6A and 6B). Between the constrictions the epidermis is up to 25 μm thick and contains the full complement of support, secretory and pigment cell-types; at the deepest point of the constrictions the epidermis can be as little as 2 μm thick with the only cellular components being apical and basal portions of support cells (Fig 7A–7C). The lamina densa underlying the constrictions tends to be thinner and to have a delaminated appearance with sub-layers splitting away from the inner side and forming tubular structures and curved sheets with curlicue-like profiles (Fig 7C and 7E). Such delamination was also observed in some epidermal regions remote from constrictions. At the deepest point of the constrictions there are prominent hemidesmosome-like junctions between basal portions of the support cells and the basement membrane (Fig 7C). At such locations there is within the lamina lucida an electron-dense plate which is linked by fine filaments on one side to the lamina densa and on the other to an area of plasmalemma adjacent to electron-dense cytoplasmic material to which are attached bundles of tonofibrils (Fig 7D). These junctions are much larger than those observed in other regions of the joint epidermis (Fig 7C).

#### Spine ligament

The spine ligament consists mainly of densely packed collagen fibres roughly parallel to the longitudinal axis of the ligament, though usually with an undulating appearance (Fig 6C and 6D). The ligament is attached to the lateral plate and spine base by means of thick, deeply penetrating fibres interconnected by branches that loop around successive layers of stereom bars (Fig 6C and 6D). As was inferred from the interruptions in the ligament insertion areas of the tubercle and spine base, the ligament does not completely enclose the joint; at the oro-proximal side there is a gap that is widest at the tubercle and narrows towards the spine base (Fig 6E).

**Fig 8:**
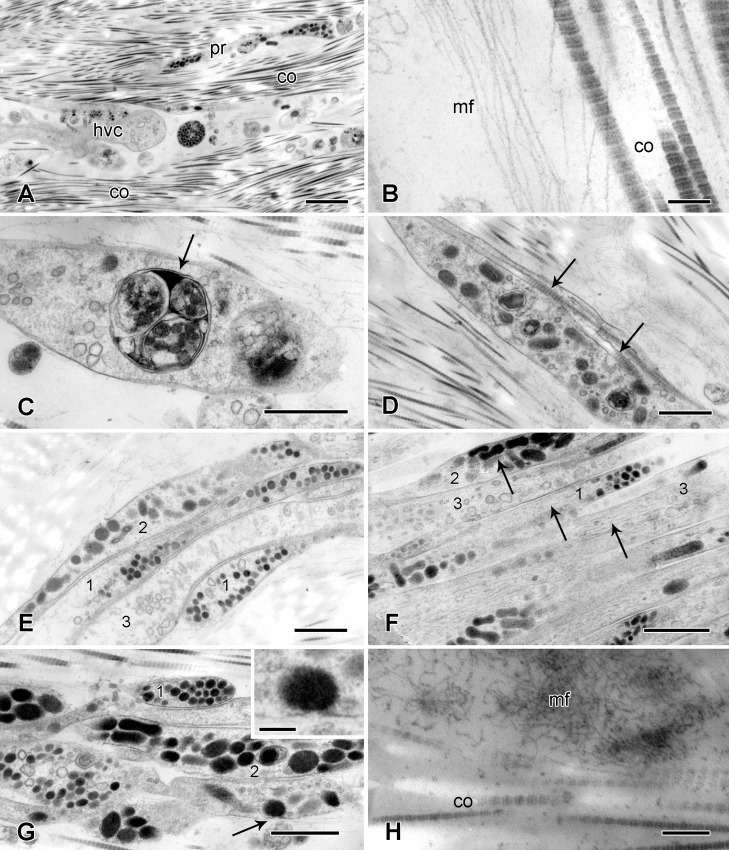
Ultrastructure of the spine ligament and interarticular connective tissue. Transmission electron micrographs. (A-G) Spine ligament. (A) Low magnification view showing the main components. Scalebar: 2 μm. (B) Edge of a fibril bundle showing collagen fibrils and microfibrils. Scalebar: 0.2 μm. (C) Cell containing secondary lysosome-like vesicle (arrow). Scalebar: 1 μm. (D) Cell that appears to have phagocytosed collagen fibrils (arrows). Scalebar: 1 μm. (E) Cluster of cell processes. 1, type 1 DCV-containing process; 2, type 2 DCV-containing process; 3, process containing only electron-lucent vesicles. Scalebar: 1 μm. (F) Cluster of cell processes. Arrows indicate microtubules. Scalebar: 1 μm. (G) Cluster of cell processes, including an omega-profile suggestive of exocytosis (arrow and inset). Scalebars: 1 μm (main panel) and 0.2 μm (inset). (H) Interarticular connective tissue, which consists of loosely arranged collagen fibrils and microfibrillar meshworks. Scalebar: 0.2 μm. co, collagen fibrils; hvc, cell containing heterogeneous vesicles; mf, microfibrils; pr, cell process containing dense-core vesicles.

Each ligament fibre is a tightly packed bundle of collagen fibrils with a mean banding periodicity of 59.6±2.2 nm (*n* = 10) and maximum diameter around 90 nm (Fig 8A and 8B). Between the fibril bundles, and aligned mostly parallel to them, there are loose arrays of microfibrils with a maximum diameter of 7–14 nm and sometimes with a beaded appearance (periodicity 26.9±4.0 nm; *n* = 5) (Fig 8B–8D).

The ligament contains a sparse scattering of nucleated cell bodies (Fig 6F), which appear to have a phagocytic function, since their cytoplasm contains many heterogeneous vesicles, some with electron-lucent contents, others containing cell debris or collagen fibrils (Fig 8A, 8C and 8D). The ligament is permeated by many cell processes aligned parallel to the collagen fibres and fibrils, which do not emanate from the fibroclast-like cells (Fig 8A). Three types of processes can be distinguished in ultrathin sections (Fig 8E–8G). All three lack a basement membrane and contain microtubules (Fig 8F). Type 1 processes contain membrane-bounded dense-core vesicles (DCVs) with profiles that are usually circular but occasionally oval, with a mean diameter of 164±19 nm; they may also have variable numbers of lucent vesicles (LVs) with a roughly circular profile and diameter similar to that of the DCVs (Table 1). Some DCVs and LVs are closely adjacent to the cell membrane (Fig 8E–8G). There was, however, no overt evidence that either undergoes exocytosis (such as the presence of omega (Ω)-profiles). Type 2 processes contain DCVs with circular to (more often) elongate-oval profiles, the latter often being constricted in the middle (“waisted”) and of mean length 450±54 nm and mean maximum width 192±31 nm. As well as close contacts between some type 2 DCVs and the cell membrane (Fig 8E–8G), two possible omega-profiles were observed (Fig 8G) (in a total of 33 sections of type 2 processes examined during this investigation). Type 3 processes lack DCVs but have LVs with a roughly circular profile and diameter similar to that of the type 1 LVs (Table 1); type 3 LVs sometimes contain a small granular inclusion of variable electron-density (Fig 8E). The three types of processes usually occur in closely packed parallel bundles that always include more than one type; in larger bundles all three types are always present. Within these bundles the cell membranes of adjacent processes tend to be separated by a gap whose width is constant for each pair of processes but varies between pairs (mean width 26.1±4.1 nm; range 18.5–29.6 nm; *n* = 18) (Fig 8E).

**Table 1:** Dimensions (in nm) of intracellular dense-core vesicles (DCVs) and lucent vesicles (LVs) of cell processes in the ligament and of cell bodies in the juxtaligamental node.

	Ligament: cell process types					Juxtaligamental node: cell body types		
	Type 1: DCVs	Type 2: DCVs		Type 1: LVs	Type 3: LVs	Type 1: DCVs	Type 2: DCVs	
	Max. diameter	Max. length	Max. width	Max. diameter	Max. diameter	Max. diameter	Max. length	Max. width
Mean	164^1,5^	449^2^	191^3^	170^4,5^	180^4^	121^1^	276^2^	171^3^
s.d.	19	54	31	34	20	14	18	25
Min.	140	350	130	122	156	100	250	140
Max.	200	520	270	252	216	150	310	220
*n*	25	20	20	12	12	18	11	11

Statistical analysis (ANOVA and Bonferonni’s post test) of pairs of means sharing the same superscript numeral indicated significant differences (*P* <0.05) for comparisons 1 and 2, but not for 3, 4 or 5. A third cell body type, which was present in the node, is not included, since it lacked both DCVs and LVs.

#### Interarticular connective tissue

In histological sections, outlines of the articular surfaces of the tubercle and spine base are discernible, even when at fixation the spine was erect and the two surfaces tightly pressed together, mainly because of the presence between the articular surfaces of fibrous connective tissue (Fig 6C–6E, 6G and 6H). The fibres in this tissue have no preferred orientation and consist of loose bundles of collagen fibrils. The fibres are widely spaced and between them are many 7 nm microfibrils, some forming large tangled meshworks (Fig 8H). No cell bodies or processes were observed in this tissue. It is attached to the articular surfaces of the tubercle and spine base by thick fibril bundles that penetrate deeply into the stereom via the sparse pores observed in SEM images (Fig 6C and 6D). The only location where articular surfaces are not separated by interarticular connective tissue (i.e. where there is direct contact between skeletal components) is the apposition between the oro-proximal region of the oral lobe of the tubercle and the distal region of the proximal condyle of the spine base (Fig 6C).

#### Juxtaligamental node

**Fig 9:**
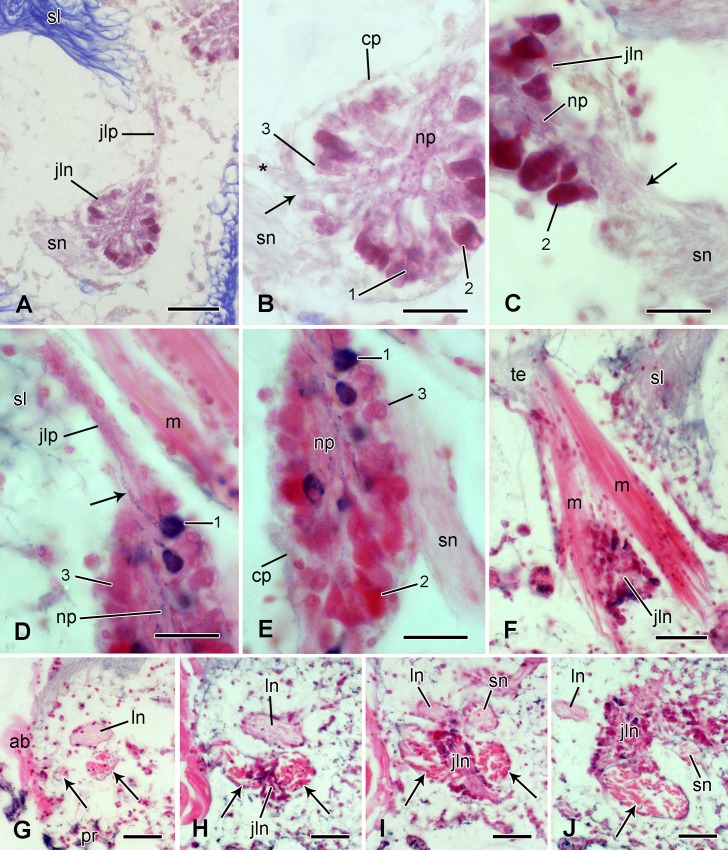
Microstructure of the juxtaligamental node and muscle. Light micrographs. (A-F) Transverse sections. (A) Overview of the anatomical relationships between the juxtaligamental node, spine ligament and spine nerve. Stained with Milligan’s trichrome (MT). Scalebar: 40 μm. (B) Juxtaligamental node shown in A. 1, 2 and 3 are the cell types described in the main text. The arrow indicates cell processes extending between the spine nerve and the node. The asterisk marks the perineurium of the spine nerve. Stained with MT. Scalebar: 20 μm. (C) Juxtaligamental node, showing continuity of a branch of the spine nerve (arrow) with the neuropil-like region. Stained with MT. Scalebar: 20 μm. (D) Juxtaligamental node, showing presence of cell type 1 processes (arrow) in the neuropil-like region and in the cluster extending to the spine ligament. Stained with Bargmann’s chrome haematoxylin and phloxine (CAHP). Scalebar: 20 μm. (E) Remainder of the same juxtaligamental node shown in D. Stained with CAHP. Scalebar: 20 μm. (F) Spine muscle: insertion into spine base at top left and insertions into lateral arm plate at bottom right. Stained with CAHP. Scalebar: 40 μm. (G-J) Horizontal sections showing the anatomical relationships between the spine muscle (arrows), lateral arm plate nerve and juxtaligamental node. The plane of the sections and orientation of the micrographs are the same as in Fig 6F–6H. Also as in Fig 6F–6H, the plane of section is progressively abradial (towards spine base). Stained with CAHP. (G) The muscle consists of two widely separated myocyte bundles (arrows). Scalebar: 40 μm. (H) The adradial edge of the juxtaligamental node is visible between the two myocyte bundles. Scalebar: 40 μm. (I) The main body of the juxtaligamental node is closely adjacent to both myocyte bundles and the spine nerve has branched from the lateral arm plate nerve. Scalebar: 40 μm. (J) The two myocyte bundles have merged to form a single muscle (arrow). Scalebar: 40 μm. ab, aboral side; cp, capsular layer; jln, juxtaligamental node; jlp, juxtaligamental cell processes; ln, lateral arm plate nerve; m, muscle; np, neuropil-like region; pr, proximal side; sl, spine ligament; sn, spine nerve; te, tendon connecting muscle to spine base.

The type 1 and 2 cell processes of the spine ligament belong to a discrete cluster of cell bodies located within the lateral plate beneath each tubercle. This is a typical ophiuroid juxtaligamental node [16,43–45] whose most prominent components are unipolar pyriform cell somata arranged circumferentially with their single processes directed centrally and contributing to nerve-like tracts that emerge from the node and extend through the lateral plate to the insertion regions of the adjacent ligament. Here the tracts subdivide and their branches pass into the ligament via the thick insertion fibres (Fig 9A–9D). There appear to be three histochemically distinct populations of pyriform cells: (1) the perikarya are granular, stained red-violet by Milligan’s trichrome (MT) and stained black by the chrome alum haematoxylin (CAH) of Bargmann's chrome alum haematoxylin and phloxine; (2) the perikarya consist mostly of an intensely acidophilic (i.e. red with MT and magenta with Bargmann’s stain) mass; the processes of both these cell-types, identifiable by their CAH-positivity and acidophilia respectively, are present in the tracts and ligament; (3) the perikarya are agranular, weakly basophilic (pale red-violet to grey-blue) with MT, and CAH-negative (Fig 9B–9E). The node is delimited by a thin outer capsule that is weakly stained by MT and is CAH-negative (Fig 9B and 9E).

**Fig 10:**
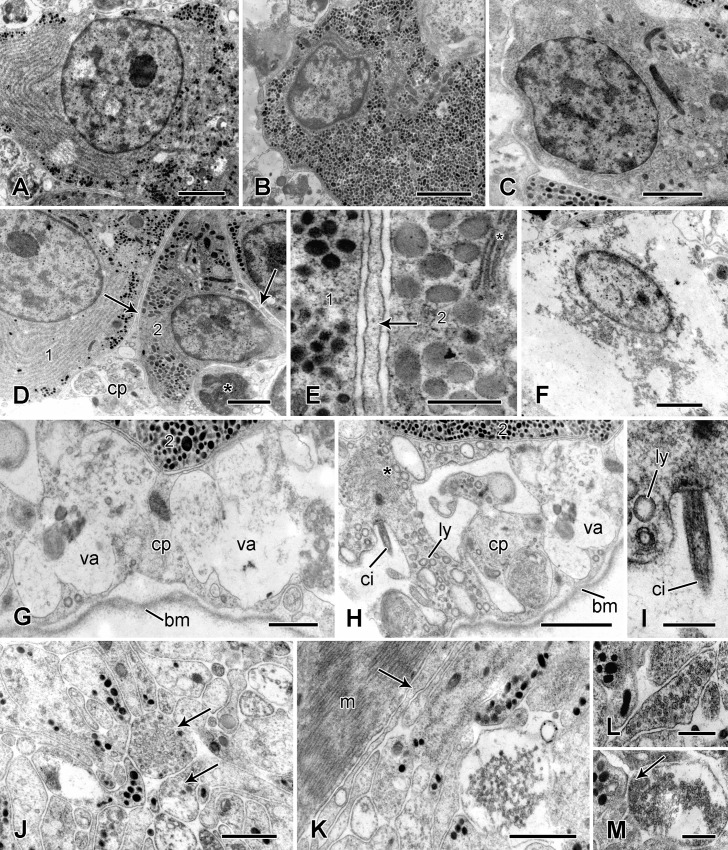
Ultrastructure of the juxtaligamental node. Transmission electron micrographs. (A) Type 1 cell body. Scalebar: 2 μm. (B) Type 2 cell body. Scalebar: 2 μm. (C) Type 3 (agranular) cell body. Scalebar: 2 μm. (D) Edge of a node, showing the capsular layer and extensions of capsular cells (arrows) separating adjacent DCV-containing cells (1, 2). The asterisk marks a capsular cell vacuole containing possible cell debris. Scalebar: 2 μm. (E) Capsular cell extension (arrow) between type 1 and 2 DCV-containing cells (1, 2). The asterisk marks a Golgi complex. Scalebar: 0.5 μm. (F) Cell undergoing degradation in the capsular layer. Scalebar: 2 μm. (G) Capsular cell with large vacuoles and basement membrane. Scalebar: 1 μm. (H) Same capsular cell as shown in G, with cilium, lysosome-like vesicles and Golgi complex (asterisk). Scalebar: 2 μm. (I) Cilium of cell shown in H. Scalebar: 0.5 μm. (J) Neuropil-like region of a juxtaligamental node, which includes profiles containing small spherical vesicles (arrows). Scalebar: 1 μm. (K) Neuropil-like region, which is separated from the spine muscle by a thin cellular partition (arrow). Scalebar: 1 μm. (L, M) Cell processes in the neuropil-like region, which contain two types of small vesicles and are adjacent to DCV-containing cell processes. That in M includes a synapse-like junction (arrow). Scalebars: 0.5 μm. bm, basement membrane; ci, cilium; cp, capsular layer; ly, lysosome-like vesicle; m, spine muscle; va, vacuole.

In ultrathin sections there are two types of pyriform cells containing large membrane-bounded DCVs with medium to high electron-opacity that are likely to correspond to the CAH-positive and acidophilic inclusions observed in the light microscope. In one type, the DCVs tend to be completely electron-opaque, their profile is circular to slightly oval with a mean diameter of 122 ± 15 nm and they form discrete aggregates adjacent to the plasmalemma. The perikaryon also contains large amounts of rough endoplasmic reticulum, mitochondria and Golgi complexes. The nucleus has a prominent, evenly stained nucleolus and a high proportion of euchromatin (Fig 10A, 10D and 10E). Since the main DCVs of these cells and those of type 1 processes have the same shape and overlapping size ranges (Table 1), it is highly likely that type 1 processes belong to these cells and they will be referred to hereafter as type 1 cells. In the second cell-type, the DCVs more often have medium electron opacity, their profile is circular to elongate-oval, with some of the oval DCVs being waisted (i.e. constricted in the middle), and they have a mean length of 276 ± 18 nm and width of 172 ± 25 nm; the DCVs are dispersed throughout the perikaryon, together with mitochondria and Golgi complexes; rough endoplasmic reticulum is much less evident in this type than in the first cell-type. The nucleus of these cells tends to have a higher proportion of heterochromatin and the nucleolus is either not visible or has irregular electron-opaque or electron-lucent inclusions (Fig 10B, 10D and 10E). Their DCV size and shape suggest that cells of the second type give rise to type 2 processes (Table 1) and they will be referred to as type 2 cells. In addition, interspersed amongst the DCV-containing cells is a third cell-type that lacks DCVs but contains much rough endoplasmic reticulum, Golgi complexes, mitochondria and a few large (diameter 700–800 nm) vesicles with heterogeneous contents (Fig 10C).

The outer capsule of the node consists of a single layer of cells and their basement membrane, which is continuous with the perineurial epithelium of the spine nerve and its branch to the node (see below) (Fig 9B). The capsular cells have cilia, which project into large intercellular spaces (Fig 10H and 10I), and they have partition-like extensions around 100 nm wide, which pass centripetally between adjacent granule-containing cells and reach the central region of the node (Fig 10D and 10E). In places the cytoplasm of the capsular cells has an unexceptional appearance and includes mitochondria, Golgi complexes and primary lysosome-like electron-lucent vesicles of variable diameter (Fig 10H). Elsewhere, however, the cell interior is filled with very large, coalescing, phagosome-like vacuoles containing heterogeneous material, much of which resembles cell debris (Fig 10G and 10H). Other indications of cell degradation include outlines of cell bodies containing only cytoplasmic remnants or isolated nuclei surrounded by sparse granular material (Fig 10F).

In the central region of the node there is a dense neuropil-like aggregation of cell processes, including DCV-containing types belonging to the pyriform cells and others, some of which are closely apposed to the former, containing small, spherical, uniformly sized vesicles (Fig 10J–10M). Two types of small vesicle are distinguishable: one has a mean diameter of 75±7 nm (*n* = 10) and contents of medium electron opacity with a slightly darker core (Fig 10L); the other, which tends to form sub-plasmalemmal clusters reminiscent of the configuration at pre-synaptic junctions, has a mean diameter of 64±7 nm (*n* = 10) and homogeneous contents of medium electron opacity (Fig 10M). The cell processes containing small vesicles are likely to be neurites: the undivided spine nerve is close to the node and, though it is mostly separated from the node by the perineurial epithelium, it emits a short branch containing cell processes that extend from the spine nerve into the neuropil-like region of the node (Fig 9B and 9C).

#### Spine muscle and its tendons

**Fig 11:**
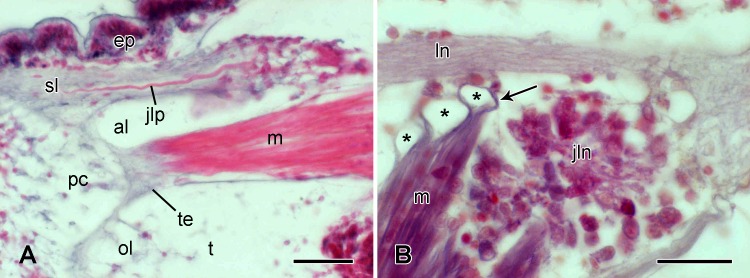
Microstructure of the spine muscle tendons. Light micrographs of transverse sections. (A) Tendon connecting the muscle to the proximal condyle of the spine base. Stained with CAHP. Scalebar: 40 μm. (B) Tendons (arrow) connecting the muscle to stereom bars (asterisks) of the lateral arm plate. Stained with MT. Scalebar: 20 μm. al, aboral lobe; ep, epidermis; jln, juxtaligamental node; jlp, juxtaligamental cell processes; ln, lateral arm plate nerve; m, muscle; ol, oral lobe; pc, proximal condyle; sl, spine ligament; t, tubercle; te, tendon.

The spine muscle consists of two bundles of myocytes with separate areas of origin within the lateral plate, which are, respectively, on the oral and aboral sides of the distal end of the juxtaligamental node (Fig 9F). The two bundles converge to form a single bundle that is attached to the proximal condyle of the spine base (Figs 6F–6H and 9F–9J). Each myocyte spans the full length of its bundle from origin to insertion. The bundles are linked to the proximal condyle of the spine base by a single compact cord of thick tendon fibres that penetrate deeply into the stereom (Fig 11A), and to the lateral plate by fine extracellular tendon fibres that loop round superficial stereom bars (Fig 11B).

**Fig 12:**
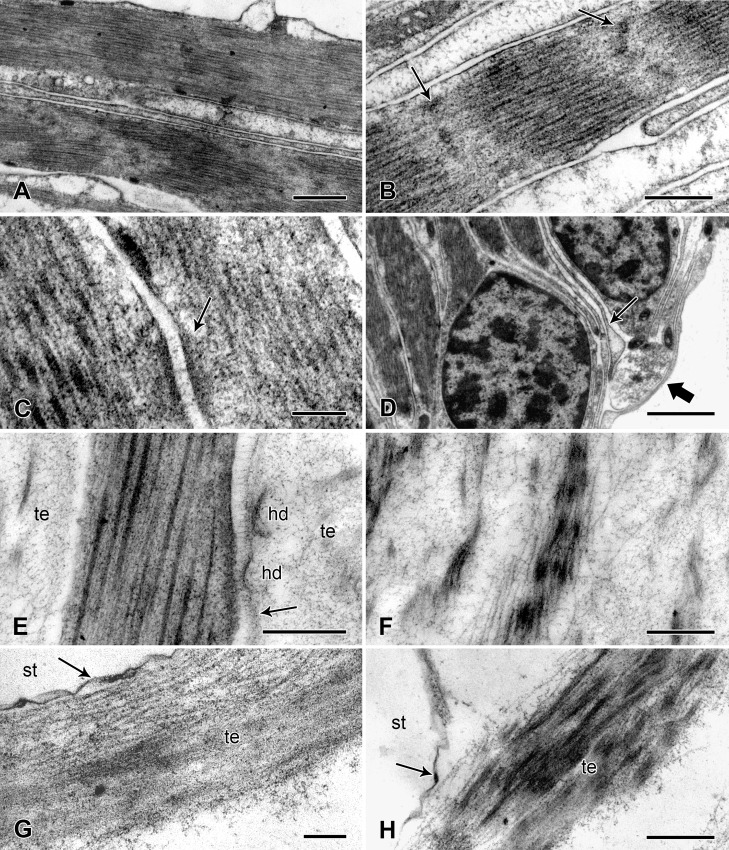
Ultrastructure of the spine muscle and tendons. Transmission electron micrographs. (A) Longitudinal section of two myocytes, showing oblique striations. Scalebar: 1 μm. (B) Longitudinal section of a myocyte. Arrows indicate Z-bodies. Scalebar: 0.5 μm. (C) Desmosome-like junctions (arrow) between two myocytes. Scalebar: 0.2 μm. (D) Myocyte extensions (thin arrow) are in contact with a cell process containing small vesicles (thick arrow). Scalebar: 2 μm. (E, F) Tendon connecting the muscle to the spine base: region adjacent to the muscle. (E) The tendon has a dense delimiting layer (arrow), which is connected to the myocytes at hemidesmosome-like junctions. Scalebar: 0.5 μm. (F) The spine base tendon includes prominent fibre-like aggregations of microfibrils. Scalebar: 0.5 μm. (G, H) Tendon connecting the muscle to the lateral arm plate: tendon loop adjacent to a stereom bar (see arrow in Fig 11B). (G) The tendon is closely adpressed against the extracellular “matrix coat” (arrow) that envelopes the calcite stereom (see [46]). Scalebar: 0.2 μm. (H) Point at which the tendon diverges from the stereom bar. Arrow indicates the “matrix coat”. Scalebar: 0.5 μm. hd, hemidesmosome-like junction; st, calcite stereom (decalcified); te, tendon.

The myocytes are obliquely striated, i.e. it can be seen in longitudinal sections that adjacent myofilaments are not lined in register but are progressively staggered across the width of each cell (Fig 12A and 12B). Thin filaments are attached to electron-dense Z-bodies (Fig 12B). The diameters of the thick and thin myofilaments are around 40 nm and 10 nm respectively. There are desmosome-like junctions between adjacent myocytes (Fig 12C) and fine myocyte extensions lacking myofilaments are in contact with cell processes containing small vesicles with a circular profile (diameter up to ca. 90 nm) and contents of medium electron density (Fig 12D).

The lateral arm plate and spine base tendons are extensions of myocyte basement membranes. Their most abundant components are microfibrils with a diameter of 8–13 nm. These are present as a loose array, but some coalesce into parallel electron-dense assemblages, which are especially prominent in the spine base tendons where they form distinct fibres (Fig 12E–12H). Adjacent to the myocytes, there is a dense delimiting layer of tendon microfibrils, which is separated from the myocyte plasmalemma by an electron-lucent gap 40–110 nm wide. There are many hemidesmosome-like junctions between the myocytes and tendons where the electron-lucent gap is bridged by fine filaments linking the delimiting tendon layer to an area of plasmalemma underlain by electron-dense cytoplasmic material (Fig 12E).

As can be seen in Fig 9F and 9H–9J, the spine muscle appears to be in contact with the juxtaligamental node. Where they are closely adjacent, myocytes and juxtaligamental cells are separated by a thin partition consisting of a single layer of cells of an unidentified type; although these are likely to be capsular cells, no basement membrane was observed (Fig 10K). No separate nerve branch innervating the muscle was found. However, the proximal insertion areas of the muscle are very close to the lateral arm plate nerve (Fig 11B); its efferent supply may reach it directly from the lateral arm plate nerve or indirectly via the juxtaligamental node.

### Spine behaviour in living animals

In undisturbed animals observed directly in holding tanks or on the sea-floor and in animals confined to small vessels of seawater and observed with a stereomicroscope, the spines were usually fully erect, i.e. they projected radially and were parallel to a plane perpendicular to the longitudinal axis of the arm, those of adjacent arm segments being arranged in precisely aligned longitudinal, comb-like rows (Fig 1). As well as the spines being fully erect, the spine joints were usually highly mobile with respect to inclination within a particular directional range: (1) the spines evinced no resistance to being brushed gently with a probe in the aboral, distal or aboro-distal directions (subsequently abbreviated to ‘aborally to distally’), although they resisted attempts to brush them in any other direction; (2) when moving arms breached the water surface, surface tension provided sufficient force to tilt the spines aborally to distally (but not in any other direction) and depress them maximally against the sides of the arms; (3) spine inclination in aboral to distal directions sometimes occurred when fully immersed arms moved rapidly through the water. Following removal of the external force causing spine inclination–whether this was due to touching with a probe, contact with the water surface or hydrodynamic drag–the spines immediately returned to the fully erect position and became precisely aligned as before.

The behaviour of spines was compared before and whilst animals were in an anaesthetised condition (resulting from immersion for at least 30 min in seawater containing 0.1% propylene phenoxetol). The spines of anaesthetised animals remained erect, but the joints showed greater mobility than before anaesthetisation, in that they showed no resistance to externally imposed inclination in any direction, including orally and proximally. After forced inclination, the spines re-erected, but it was consistently seen (no matter the direction in which spines had been tilted) that recovery occurred more slowly than in the unanaesthetised state and was frequently incomplete, i.e. the spine did not reach the fully erect position.

Joint mobility was investigated in autotomised arms. Arms were induced to autotomise by compressing them near the disc with forceps and joint mobility was regularly assessed by bringing the arm into contact with the water surface. In arms undergoing this treatment the spine joints remained highly mobile before autotomy occurred, as indicated by the aboral to distal inclination of the spines when they breached the water surface, but after the arm was detached the joints became less mobile, i.e. the spines were not depressed by contact with the water surface. The spines of autotomised arms also showed greater resistance to stroking with a probe: in some cases the spines could still be fully depressed when stroked aborally to distally, but greater force was required to achieve this than before anaesthetisation; in other cases such attempts to depress the spines were resisted to the extent that spines broke without any significant inclination occurring. This state of reduced mobility persisted in the spine joints of autotomised arm pieces that were immersed in anaesthetic solution for 30 min.

**Fig 13:**
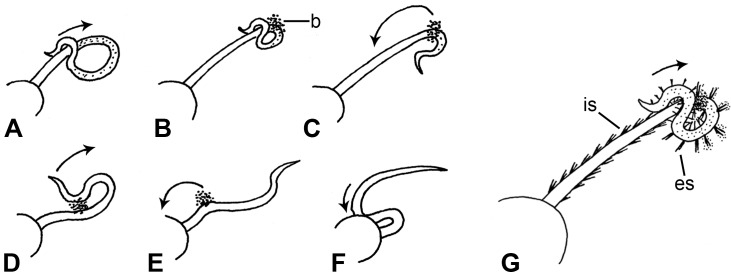
Arm self-wiping behaviour of *O*. *nigra*. Drawings (not to scale) illustrating the response of arms to particles settling on their aboral surface. (A-F) A complete sequence of bolus formation and transfer to the mouth. (G) The dragging action of the arm-loop inclines spines distally and aboro-distally. b, bolus; es, fully erect spines; is, inclined spines.

An indication of the functional significance of spine inclination was provided by animals performing a particular type of feeding behaviour in holding tanks, which has not been described previously and which was observed when plankton or detritus particles settled on the aboral surface of their arms. The distal part of an arm was draped over its proximal region to form an inverted U-shaped loop; this loop was then dragged distally along the arm, thereby wiping particles from the aboral surface and amassing them into a bolus, which was transferred by lateral flexion of the arm initially to a position on the arm closer to the disc and then to the mouth (Fig 13A–13F). The relevance of this food-collecting mechanism to spine movements is that the dragging action of the arm loop depressed the spines in distal or aboro-distal directions, which both permitted closer contact between the loop and the aboral arm surface and enabled it to brush particles from the spines (Fig 13G). This behaviour was seen in stationary animals and in crawling animals; in the latter case, one arm collected particles by the self-wiping method whilst the other four were involved in locomotion.

### Spine tagging experiment

**Fig 14:**
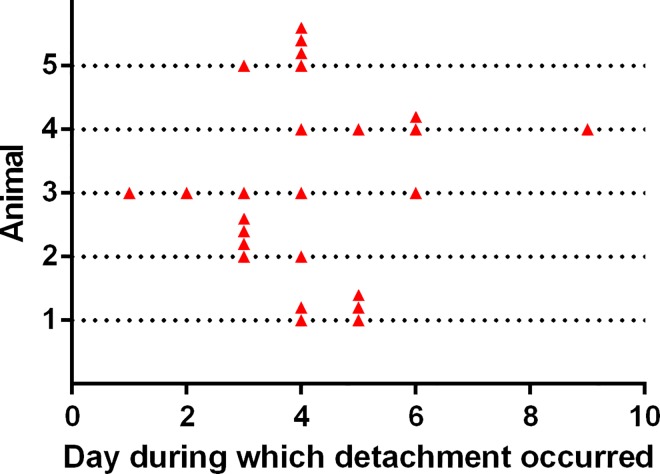
Results of the spine tagging experiment. Each dotted horizontal line represents one animal and the symbols indicate the day on which tagged spines were detached.

The animals jettisoned all 25 tags, 24 through detachment of arm spines, and one by indeterminable means (since no spines were attached to it). The shortest interval between tag attachment and loss was one day, the longest was nine days, and the mode was four days (Fig 14). Although when setting up the experiment care had been taken to ensure that each tag was cemented to only one spine, only three detached tags had a single spine adhering to them; the other 21 tags had two to eight spines attached (mean ± s.d. 3.7 ± 2.1). Of the total of 88 spines adhering to tags, 85 had become disengaged at the joint and two had broken off through the shaft.

### Experiment on individual spine joints

Seventeen spine preparations were tested. When the tensile load was applied to individual spines, there was an initial phase of rapidly decelerating extension, which lasted 21 ± 12 s (mean ± s.d.) and led to a phase of extension at a roughly constant rate (mean ± s.d. 0.016 ± 0.014 mm min^-1^). Preparations extending at a constant rate were left undisturbed for up to 10 min before stimulation by elevated [K^+^]_o_ (0.56 mol l^-1^ KCl or seawater containing 100 mmol l^-1^ K^+^). Six of the 17 preparations showed no change in extension rate within three min of the start of treatment and the other 11 preparations showed an increase in extension rate within three min (mean response time 54 ± 46 s) (Fig 2B). In eight of the 11 potassium-responsive preparations the increase in extension rate culminated in rupture of the spine joint and detachment of the spine (mean response time 94 ± 45 s). The other three potassium-responsive preparations were observed for up to four min and then the test was terminated.

## Discussion

### Preamble

Each of the following sections deals with a separate structural entity and addresses morphological and, where appropriate, mechanical aspects, the latter incorporating information obtained from the direct observation of spine behaviour and from the two experiments. An overview of spine joint functioning is provided in the ‘Conclusions’.

### Skeletal components

There appears to have been no previous functional analysis of the arm spine joint of any ophiuroid, with the exception of that of *Ophiopholis aculeata* (L., 1767) contained in an unpublished thesis [47] micrographs from which are included in Byrne’s review [16]. Scanning electron micrographs of the skeletal components of the joint, i.e. of the lateral arm plates and spine base, of *O*. *nigra* have been published [18,19,42], but unaccompanied by any consideration of their functional implications.

**Fig 15:**
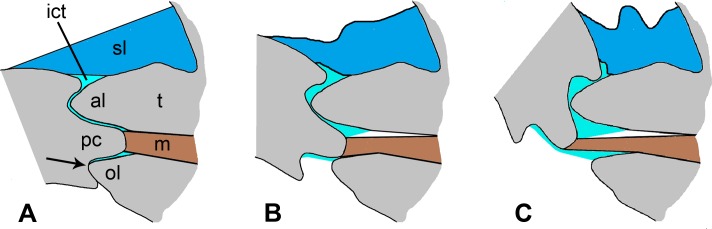
Diagrams illustrating aboral inclination of a spine. (A) Spine fully erect. Accurate representation of the articular surfaces based on a transverse histological section. No ligament is present at the oral side of the joint, because the plane of the section passes through the gap in oral region of the ligament. The arrow indicates the apposition where interarticular connective tissue is lacking and the spine base and tubercle are in direct contact. (B, C) Two stages in the aboral inclination of the spine, assuming minimal separation between the articular surfaces. The aboral portion of the spine ligament is compressed; the articular surfaces do not maintain a close fit; and the muscle is stretched. al, aboral lobe of tubercle; ict, interarticular connective tissue; m, muscle; ol, oral lobe of tubercle; pc, proximal condyle of spine base; sl, aboral portion of spine ligament; t, tubercle.

The articular surfaces of the lateral plate tubercle and the spine base of *O*. *nigra* consist of (1) on the aboral side, a reniform apposition between the convex aboral lobe of the tubercle and the concavity of the spine base, and (2) on the oral side, a mechanical stop formed by the proximal and distal condyles of the spine, which fit tightly between the aboral and oral lobes of the tubercle when the spine is fully erect (Fig 15). The mechanical stop function of the spine base condyles is indicated both by the peg-in-socket relationship between them and the tubercle lobes and by the presence of abrasions on the distal region of the proximal condyle, which sits against the oral lobe of the tubercle, but only when the spine is fully erect (Figs 5G, 6C and 15). The functional significance of the reniform apposition is less obvious.

The skeletal articulations of animals can be broadly classified as immobile joints (synarthroses) or mobile joints (diarthroses) [48]. Since the inclination of the spines can change in unanaesthetised living *O*. *nigra*, the articulation of the arm spine with the lateral arm plate can clearly function as a mobile joint. There are two main types of mobile animal joints: (1) sliding joints, which transfer loads directly between skeletal components that are in direct contact with each other, and (2) flexible joints, which transfer loads indirectly between skeletal components that are not in direct contact but are connected by an intercalated pliable material [49–51]. The shape of the individual articular surfaces forming the reniform apposition suggests that this is a sliding joint in which the two surfaces remain in close contact during spine inclination. However, Fig 15, which illustrates the relationship between the articular surfaces during inclination of the spine in the aboral direction, shows that the articular surfaces, despite being closely fitting when the spine is fully erect, cannot maintain this close fit when the spine inclines. In addition, since the articular surfaces of the reniform apposition are separated at all times by interarticular connective tissue, they are never in direct contact. These morphological considerations, together with the laxity that is the usual condition of the joint and in which inclination of the spine is not restricted to a single plane but occurs through a range of directions from the aboral to the distal, suggest that the articulation functions usually as a flexible joint. By way of contrast, the arm spine articulation of *O*. *aculeata* appears to be a sliding joint, which allows movement in only a single fixed plane [47].

Whilst the spine joint of *O*. *nigra* is usually lax, its mobility, as signified by its resistance to external forces applied to the spine, is variable and the joint can be immobilised to the extent that attempts to depress the spine result in its breakage without causing significant joint motion, as was observed in autotomised arms. The spine joint of *O*. *aculeata* also shows variable mobility, including an immobilised condition in which the spines (which are more robust than those of *O*. *nigra*) can be moved passively, but not without damaging the soft tissues [47]. Thus these joints can operate as both mobile diarthroses and immobile synarthroses. Such functionally versatile joints occur in other echinoderms (spine joints of echinoids: [5]) and in vertebrates (e.g. the fin spine joints of certain fish: [49,52]). Discussion of the physiological and mechanical bases of the functional shift shown by the ophiuroid joints is provided below.

The perforations occurring in the calcite stereom of the reniform articular surfaces of *O*. *nigra* accommodate the fibres that anchor the interarticular connective tissue to the aboral lobe of the tubercle and to the concavity of the spine base. Perforate stereom is also present and associated with interarticular connective tissue at the articular surfaces of certain other ophiuroid joints, including the radial and interradial joints of the mouth-frame, in which, as in the arm spine joint, the articular surfaces separate during joint motion ([53]; I.C. Wilkie, personal observations). The presence of perforate stereom in the aboral tubercle lobe and spine base concavity shows a wide taxonomic distribution across the Ophiuroidea, the main exceptions being members of the closely related families Amphiuridae, Ophiactidae and Ophiotrichidae, which, like *O*. *aculeata* (an ophiactid), have a distinctive joint morphology characterised by articular tubercles consisting of two roughly parallel ridges of imperforate stereom [17–19]. This joint type lacks interarticular connective tissue, assuming that *O*. *aculeata* is representative [47].

### Circumarticular epidermis

In contrast to most of the epidermis of *O*. *nigra*, that overlying the arm spine joint (and the joints between the outer skeletal plates: [41]) is not directly attached to endoskeletal tissue. It overlies the spine ligament from which it is separated by a fluid-filled cavity and it is folded circumferentially, as is also the case in *O*. *aculeata* [47]. These features probably ensure that the epidermis neither restricts spine movements nor is subjected to excessive tension by them, especially on the extension side of the joints when spines are fully recumbent.

Epidermal folding is facilitated by circumferential constrictions at which the thickness of both the cellular layer and the extracellular lamina densa is reduced, as has been observed at the base of human skin wrinkles [54]. The support cells of the epidermis are connected to the lamina densa at structural complexes whose morphology is identical to that of mammalian hemidesmosomes, in that they include components corresponding to mammalian anchoring filaments, sub-basal dense plate (both components located within the lamina lucida) and cytoplasmic plaque linking the plasma membrane to intracellular tonofibrils [55]. Those hemidesmosomes closest to, and on either side of, the flexion plane at the deepest level of the constrictions were much larger than those located at more superficial levels, which presumably is an adaptation for resisting the greater shear and tensile forces occurring between the support cells and basement membrane near the flexion plane when the epidermis is stretched and compressed by spine movements.

### Spine ligament

Regarding its histological organisation and the topology of its insertions into the lateral arm plate and spine base, the spine ligament resembles the intervertebral ligament of *O*. *nigra* and other parallel-fibred echinoderm ligaments [12,38,56,57]. The ultrastructure of its collagen fibres is also typical for echinoderm connective tissue, in that they consist of bundles of cross-banded collagen fibrils interspersed with microfibrils. Evidence from both holothurians and echinoids indicates that the microfibrils contain a protein resembling the fibrillin of mammalian elastin-associated microfibrils [33,58]; they also show long-range elasticity [59].

The spine ligament contains two categories of cellular components: nucleated cell bodies and cell processes belonging to somata located outside the ligament. The former have been observed in all echinoderm fibrous connective tissue structures [32] and always are sparse and show evidence for active phagocytosis, such as secondary lysosome-like vesicles and envacuolated collagen fibrils. The absence of cells within the ligament that could be fibroblasts is also typical of echinoderm connective tissue [60]. Fibroblasts responsible for age-related growth of the ligament may be included amongst the large population of cells within stereom spaces adjacent to the ligament insertions, which were not examined in this investigation. However, both the scarcity of those nucleated cells with an apparent *fibroclastic* function and the absence from the body of the ligament of possible *fibroblasts* suggest that it has a very low turnover rate, which is not surprising in the light of evidence that adult mammalian tendon demonstrates almost no turnover [61].

The cell processes that permeate the ligament include two types containing large dense-core vesicles (DCVs). Such cell processes are a prominent component of every echinoderm mutable collagenous structure that has been investigated [32] and are absent from the small number of echinoderm collagenous structures that have been shown to be non-mutable [62–64]. The functional significance of those in the spine ligament is discussed below under ‘Juxtaligamental components’.

The spine is linked to the tubercle by several soft tissue structures: the epidermis, ligament, interarticular connective tissue, muscle and major spine nerves. As a consequence of its composition, disposition and relative cross-sectional area, the ligament must be by far the dominant determinant of the passive mechanical behaviour of the joint. The radial thickness of the ligament varies: it is greatest on the aboral and distal sides and least on oral and proximal sides, there being an actual gap in its oro-proximal region. The ligament is therefore adapted to maximise resistance to spine inclination in oral and proximal directions and minimise resistance to inclination in aboral and distal directions. This accords with the usual behaviour of spines in living animals, which showed no resistance to external forces depressing the spines in aboral to distal directions, but resisted forces acting in other directions. The ligament may also assist in re-erecting inclined spines, since this occurred in anaesthetised animals (in which the spine muscles would be incapacitated), though more slowly and less completely than before anaesthetisation. This role most likely depends on the elastic recoil of the oro-proximal region of the ligament, which would be stretched by aboral to distal inclination of the spine, and the required strain energy storage may be a function of the microfibrillar component of the ligament. Since the aboro-distal region of the ligament is compressed during aboral to distal spine inclination (see Fig 15B and 15C), it is also possible that re-erection is assisted by the elastic expansion of this region.

Given that the ligament is the mechanically dominant soft tissue component linking the tubercle to the spine, it is highly likely that the variable resistance of the joint to forces tending to rotate the spine aborally to distally results largely from the variable resistance of the ligament to deformation, which implies that the ligament is mutable collagenous tissue (MCT). Further evidence for this was provided by the tagging experiment and the experiment on individual joints. The tagging experiment demonstrated that *O*. *nigra* has the capacity to discard arm spines, a process that can occur only through the drastic weakening of the ligament. Such a loss of tensile strength is undergone by the intervertebral ligament of *O*. *nigra* during arm autotomy [38,65,66]. However, arm autotomy is achieved within a timescale of around 1 s after the onset of stimulation [65], whereas spine loss took up to nine days. This difference may be due to the low intensity of irritation caused by the presence of a tag, but more probably signifies that spine loss is not a true autotomy process but an example of opportunistic self-detachment [66], which can be achieved not because the joint is adapted for autotomy but because it is held together by MCT, the primary function of which is to modulate joint mobility. It has been noted that echinoids also tend to drop tagged spines [67], which is another example of opportunistic self-detachment that is possible because the echinoid spine ligament (or “catch-apparatus”) consists of MCT [4,5,11].

When individual joints were subjected to a constant tensile load, the resulting extension curves were typical of those produced by fibrous connective tissue structures [68], confirming that they reflect mainly the behaviour of the spine ligament. They comprised a first phase of rapid extension decelerating to a second phase of extension at a roughly constant rate. The former is usually attributed to the straightening of initially wavy or crimped collagen fibres and the consequent transfer of load to the fibres, and the latter to the elongation of the loaded fibres through slippage between their constituent fibrils [68–70]. The stimulation of preparations with elevated [K^+^]_o_ was usually followed by an increase in extension rate (indicating a reduction in ligament viscosity) and rupture of the joint, i.e. of the ligament. Responsiveness to elevated [K^+^]_o_ characterises all isolated MCT preparations [32] and results from the depolarisation of effector cells and/or neural pathways involved in the control of MCT mechanical properties (see e.g. [71]). Non-mutable echinoderm collagenous structures are either unresponsive or weakly responsive (due to non-neurally mediated physicochemical effects) [62,64].

A range of evidence thus indicates that the spine ligament consists of MCT, as was concluded by Fuhrmann [47] with respect to the spine ligament of *Ophiopholis aculeata*, and that its variable resistance to deformation underpins the ability of the joint to switch between synarthrotic and diarthrotic states: when the ligament is stiffened (in a high viscosity condition), the joint is immobilised; when the ligament is destiffened (in a low viscosity condition), it permits the joint to be lax with respect to aboral to distal inclination, though still preventing inclination in other directions (perhaps because the aboro-distal region of the ligament undergoes a smaller drop in mechanical resistance than the rest of the ligament).

### Interarticular connective tissue

Although the interarticular connective tissue (ICT) and ligament consist of ultrastructurally similar extracellular components–collagen fibrils and microfibrils, their microstructural organisation is very different. In contrast to the densely packed, parallel arrangement of fibres in the ligament, those in the ICT are widely separated and show no preferred orientation; the latter are also accompanied by more abundant microfibrils that form larger aggregations. These features imply (1) that the ICT is a highly deformable, isotropic material that can easily accommodate the large changes in the shape and volume of the interarticular spaces that occur during spine inclination (Fig 15A–15C) and (2) that it is not adapted to transfer force between the spine and tubercle or to limit the amount by which the articular surfaces can separate. The absence of any cellular elements indicates that it is not a mutable collagenous structure and therefore that it is unlikely to contribute to the regulation of joint mobility. A possible role of the ICT is to reduce the frictional forces between the articular surfaces during joint motion: the lower the friction between these surfaces, the lower the force required to depress the spine, i.e. the more lax the joint. This would be particularly relevant to the mechanical stop formed by the spine base condyles and the gap between the tubercle lobes. If there were no ICT and the articular surfaces were in direct contact, there might be a tendency for the condyles to get jammed between the tubercle lobes when the spine was subjected to forces acting to rotate it aborally to distally, thereby compromising joint laxity. This is the principle of the friction-stop mechanism that locks fish fin spines in the erect position [49,52]. It is thus proposed that the ICT facilitates joint motion, including disengagement of the mechanical stop, by acting effectively as a lubricant.

There is ICT consisting of radially orientated fibres at the joint between the pedicels and primary aboral spines of the asteroid *Acanthaster planci* (L., 1758) [12]; the ultrastructure of this specific tissue has not been described. ICT is not present in the spine joints of the echinoids *Echinus esculentus* (L., 1758) and *Psammechinus miliaris* (P.L.S. Müller, 1771) (I.C. Wilkie, personal observations). However, the joint between the proximal end of the stalk of echinoid ophiocephalous pedicellariae and the test tubercle includes interarticular extracellular material (*Arbacia lixula* (L., 1758): [72]; *E*. *esculentus* and *P*. *miliaris*: I.C. Wilkie, personal observations). In *A*. *lixula* this is acellular and consists of bundles of 10 nm microfibrils; it may function as an elastic cushion [72].

### Juxtaligamental components

The term “juxtaligamental cell” was introduced to signify neurosecretory-like, large dense-core vesicle-containing cells that were found in association with the MCT firstly of ophiuroids and subsequently of all echinoderm classes. In ophiuroids (but in no other echinoderms) juxtaligamental cell somata are incorporated into a system of ganglion-like “nodes” located outside, but usually closely adjacent to, collagenous structures, each node supplying a specific collagenous structure with DCV-containing cell processes and receiving hyponeural innervation [16,43,44].

The juxtaligamental node of the spine ligament of *O*. *nigra* resembles closely those associated with the intervertebral ligament of this species: like them it consists of a cluster of cell bodies the most prominent of which contain large DCVs and are pyriform with their single process extending into a central neuropil-like region and contributing to tracts of processes that project into adjacent ligaments; the cell cluster is delimited by a single layer of cells that are ciliated, have sheet-like extensions that separate the pyriform cell bodies and contain heterogeneous vesicles and vacuoles; and they are connected to nerve branches whose neurites contribute to the neuropil-like region [38,43]. Since all of these features are shown also by the juxtaligamental node of the arm spine ligament of *Amphipholis kochii* Lütken, 1872 ([44]; V.S. Mashanov, unpublished observations), which is phylogenetically remote from *O*. *nigra* [20], the microstructure and anatomical relationships of the intervertebral and spine ligament nodes appear to be ancestral and conserved across the Ophiuroidea.

Three pyriform cell-types were distinguishable by light microscopy (CAH-positive, acidophilic and weakly basophilic) and transmission electron microscope (type 1 with circular DCVs of diameter up to 150 nm, type 2 with ellipsoid DCVs up to 310 × 220 nm and an agranular type). Evidence from two separate sources indicates that the acidophilic cells correspond to type 2 cells: (1) in *O*. *nigra* the “autotomy tendons”, whose destabilisation permits detachment of the intervertebral muscles, are associated with only one type of juxtaligamental cell process, which is acidophilic and contains DCVs in the larger size range (diameter up to 350 nm: [62]); (2) the compass depressor ligament of the echinoid *Paracentrotus lividus* (Lamarck, 1816) contains juxtaligamental cell bodies and processes of only one type, which again is acidophilic and contains DCVs in the larger size range (diameter and length up to 280 nm and 370 nm respectively: [57]). With regard to cytoplasmic components, chrome alum haematoxylin (CAH) stains specifically rough endoplasmic reticulum and certain types of secretory granules [73]. Light microscopy revealed that intensely CAH-positive processes extend into the spine ligament. Since transmission electron microscopy showed that type 1 and type 3 cell processes within the spine ligament lack endoplasmic reticulum and that type 1, but not type 3, processes contain DCVs, it is reasonable to infer that type 1 processes (and their cell bodies) are CAH-positive.

Type 3 processes may belong to agranular cell bodies in the node, which may be conventional neurons. However, it is notable that (1) the mean sizes of their lucent vesicles (LVs) and of the LVs present in type 1 processes do not differ significantly, and (2) the mean sizes of type 1 LVs and DCVs are also not significantly different. It is therefore possible that the LVs of type 1 processes are DCVs depleted of their electron-dense contents and that type 3 processes are type 1 processes, or regions of type 1 processes, in which all the DCVs are depleted. Depletion of DCVs (resulting in “vesicle ghosts”) unaccompanied by obvious ultrastructural indicators of exocytosis (such as fusion of “ghosts” with the plasma membrane) has been correlated with the release of neuropeptides in the mammalian hypothalamus [74] and is conducive to the “kiss and run” or “direct retrieval” model of neuropeptide exocytosis, in which DCVs fuse transiently with the plasma membrane, retaining their shape after discharging their cargo and becoming available for re-use [75,76]. Type 1 DCVs are within the size-range of the peptidergic DCVs of other phyla (ca. 50–300 nm: see, e.g. [74,77–79]). The affinity of type 1 cells for CAH is further evidence that they are peptidergic: the peptidergic neurons of other phyla are stained by CAH due to its affinity for the sulphydryl groups of the cysteine-rich carrier proteins, or neurophysins [78,80], that facilitate the packaging of peptides into, and their secretion from, DCVs [81]. Neuropeptide-neurophysin complexes are expressed widely in vertebrates and invertebrates [77,81–83] and particularly relevant is the recent discovery of neuropeptide precursors with a C-terminal neurophysin domain in echinoderms, including the ophiuroid *Ophionotus victoriae* Bell, 1902 [84]. A precedent for the innervation of echinoderm MCT by peptidergic neurons is provided by the dermis of the holothurian *Apostichopus japonicus* (Selenka, 1867) which contains a population of nerve fibres immunoreactive to antibodies against the bioactive peptide stichopin [85]. Furthermore, other peptides–holokinins 1 and 2 and NGIWYamide–directly affect the stiffness of *A*. *japonicus* dermis, suggesting that peptidergic systems may have a widespread role in the control of MCT tensility [86,87].

The close apposition of type 1 and 2 processes within the spine ligament implies a functional relationship. Most mutable collagenous structures are supplied with two types of cell processes containing DCVs with roughly the same respective shapes and sizes as those of *O*. *nigra* and it has been speculated that this represents a reciprocal effector system, one type stabilising the ECM and the other destabilising it [32,88,89]. Type 2 cells, like type 1, are neurosecretory neurons, as is evident from their cytology (particularly their large DCVs and microtubules) and by the immunoreactivity of type 2-like cells in the compass depressor ligament of the sea-urchin *Paracentrotus lividus* to antibody 1E11 whose antigen is synaptotagmin B, a marker for echinoderm nerve cells (A. Barbaglio, unpublished observations; [90]). However, the proteins stiparin and *C*-tensilin, which directly stiffen holothurian dermis, have been immunolocalised to the DCVs of type 2-like cells in the dermis ([91]; D.R. Keene and J.A. Trotter, unpublished observations), suggesting that type 2 cells have an effector role. Whilst the most parsimonious hypothesis would thus be that type 2 cells directly alter ECM tensility and type 1 cells regulate this activity, the functions of these cells may be more complex. In recent years it has emerged that the DCVs of neurosecretory neurons, including peptide-secreting types, handle a diversity of molecules that are involved in a wide range of biological processes, including ECM degradation [76,92]. It is therefore possible that type 1 and type 2 cells have both effector and regulatory functions.

As in the nodes of other ophiuroid ligaments [43], the juxtaligamental perikarya of the spine ligament node (of *O*. *nigra* and other ophiuroids: [44,93]; V.S. Mashanov, unpublished observations) are in intimate contact with the cells of the capsular epithelium. Their topological relationship with the juxtaligamental perikarya and their ultrastructure indicate that the capsular cells are a type of neuroglia: (1) partition-like extensions of the capsular cells separate adjacent juxtaligamental perikarya, which resembles the compartmentalisation of neurons by “packet” glial cells in leech ganglia [94]; (2) the presence in the cells of heterogeneous phagosome-like vacuoles whose contents include cell debris suggests they have a microglia-like scavenger function [95], as has been noted for cells with a similar ultrastructure associated with the peripheral nerves of another ophiuroid [16]. In their description of the spine ligament node of *Amphipholis squamata* (Delle Chiaje, 1828), Deheyn et al. [93] referred to the capsular cells as “type D” cells, which, like those of *O*. *nigra*, are ciliated, contain heterogeneous vacuoles and compartmentalise DCV-containing cells [93,96]. In addition they were shown to be the source of bioluminescence in *A*. *squamata*, light production by isolated type D cells *in vitro* being correlated with ultrastructural changes in specialised cytoplasmic organelles [96]. It is apparent that echinoderm gliocytes display a wider morphological and functional diversity than previously realised [97] and that juxtaligamental nodes can be involved in processes other than MCT control (as is further illustrated below).

### Spine muscle and its tendons

Although the arm spine joints of all ophiuroids accommodate an erector muscle, the relative size of the muscle and its position in relation to other joint components vary widely across the class [17,20]. Information is available on the ultrastructure of the spine muscles of only two other species–*Ophiothrix fragilis* (Abildgaard in O.F. Müller, 1789) and *Amphipholis squamata*, both of which were regarded as being smooth [14,16], in contrast to the obliquely striated muscle of *O*. *nigra*. Elsewhere in the Ophiuroidea the intervertebral muscles of one species and the tube-foot muscle of three others have been found to be obliquely striated [98,99]. Muscles with oblique striations have evolved independently several times within the Metazoa (and within the Echinodermata), probably for different functional reasons [100,101]. One suggestion is that oblique striations are an adaptation for the isotonic contraction of extensible muscles [102]. This might apply to the spine muscle of *O*. *nigra*, since, as is demonstrated in Fig 15, its starting length varies with the degree of spine inclination.

The two myocyte bundles that constitute the muscle are contiguous with the juxtaligamental node and are not separated from it by a basement membrane. The close proximity of the spine muscle to juxtaligamental cells has also been noted in *Amphipholis squamata* [16]. These observations, together with the finding that extensions of the intervertebral muscle cells of *O*. *nigra* project into juxtaligamental nodes associated with the intervertebral ligaments [103], suggest that juxtaligamental nodes can have a role in the control and coordination of muscle activity as well as changes in MCT tensility. Such coordination is obviously crucial in mechanical systems where posture is maintained by MCT stiffening and active movement is accomplished by muscle contraction [11].

The spine muscle tendons of *O*. *nigra* are continuous with the basement membrane of the muscle cells, as has been reported for the spine muscle tendons of *Ophiothrix fragilis* and the intervertebral muscle tendons of *O*. *fragilis* and *O*. *nigra* [14,62]. The spine muscle tendons of *O*. *nigra* are distinctive in having a more complex organisation than that of the others, which consist mainly of a fairly homogeneous parallel array of unstriated microfibrils. In the spine muscle tendons of *O*. *nigra*, microfibrils form parallel assemblages of various types, including longitudinally aligned fibres that may serve to increase the tensile strength and stiffness of the tendons. The ultrastructural features of the hemidesmosome-like tendon-myocyte junctions described herein resemble closely those of other tendon-myocyte junctions in *O*. *nigra* and *O*. *fragilis* [14,62].

The eccentric location of the muscle at the oro-proximal edge of the lateral plate tubercle indicates that it is adapted primarily to re-erect the spine after is has inclined in the preferred aboral to distal range of directions. Since, as discussed previously, passive elastic recoil of the ligament achieves partial spine erection when the muscle is anaesthetised, it seems that contraction of the muscle may serve mainly to complete the erection process by pulling together, and ensuring close contact between, the articular surfaces of the spine base and tubercle. The small amount of information that is available on the spine movements of other ophiuroid species demonstrates that there is variation in the functional significance of spine muscle contraction. Whereas the erect position is the normal resting state for the spines of *O*. *nigra* and the role of the muscle is to return the spine to this position after it has been depressed by external forces, in *Ophiopsammus maculata* (Verrill, 1869) the short broad spines are normally adpressed closely against the lateral arm plates and spine muscle contraction brings about temporary spine erection, for example during locomotion [104].

## Conclusions—overview of spine joint functioning

The arm spine joint of *O*. *nigra* is normally a mobile diarthrosis and operates as a flexible joint in which the articular surfaces of the spine base and lateral plate tubercle are separated by compliant interarticular connective tissue. The articular surfaces constitute a reniform apposition and a peg-in-socket mechanical stop, a topology that is adapted primarily to stabilise the spine in the erect position rather than to facilitate motion between the articular surfaces. In certain circumstances erect spines can become completely immobilised, in which state the joint functions as a rigid synarthrosis. The physiological basis of the functional shift between diarthrotic and synarthrotic conditions is the capacity of the spine ligament to undergo reversible changes in tensile properties: when the joint is mobile, the ligament is destiffened; joint immobility results from the stiffening of the ligament.

Spine movements are facilitated by the presence of circumferential wrinkles in the epidermis that surrounds the joint and possibly by the interarticular connective tissue, which may reduce frictional forces between the articular surfaces. Spine inclination results only from the action of forces external to the joint and the re-erection of inclined spines is achieved partly by the passive elastic recoil of the spine ligament and partly by contraction of the spine muscle.

The role of the juxtaligamental nodes in joint functioning includes the direct modulation of the tensile properties of the spine ligament by their neurosecretory neurons (juxtaligamental cells), though the cytological and molecular mechanisms by which this is achieved in this or any other mutable collagenous structure have yet to be identified. Ultrastructural features of the nodes, particularly the compartmentalisation of the juxtaligamental cells by the glia-like capsular epithelium and their central neuropil-like regions that receive direct innervation, strongly suggest that they have an integrative role and may coordinate the effector systems of the spine joint.

It is reasonable to assume that the above functional overview applies at least broadly to the arm spine joints of all ophiuroids that have the same spine base and tubercle morphology as that of *O*. *nigra*, which is the case for most species currently assigned to the families Ophiacanthidae and Ophiocomidae [17–19]. However, there is wide variation in arm spine joint skeletal morphology across the class [17–20], which must be paralleled by differences in the morphology, anatomical relations and biomechanical roles of the soft tissue components. Hopefully this investigation will stimulate morphological and functional analyses of other spine joint types, particularly where the spines have specialised functions, such as those of certain amphiurids that support respiratory fringes assembled from substratum material [105] and the feather-spines that facilitate swimming in *Ophiernus adspersus* Lyman, 1883 [15]. This would obviously enrich our knowledge of the biology of living ophiuroids, but also has palaeontological applications. Isolated ophiuroid lateral arm plates are common microfossils, which have long been used for species identification and new taxon creation and which recently have been shown to have relevance for phylogenetic investigations [19,20]. The morphology of the spine joint tubercles on these microfossils has the potential to provide information on spine functioning and functions in extinct forms.
